# Supplementary material for: Patterns of adherence to home blood pressure monitoring among men and women in the Electronic Framingham Heart Study
Source: PLoS One. 2025 Dec 17;20(12):e0337284. doi: 10.1371/journal.pone.0337284 (PMC12711059; doi:10.1371/journal.pone.0337284)
Supplement: S1 Table — (DOCX) [file pone.0337284.s001.docx]

**Supporting information**

**S1 Table. Stratified multivariable logistic regression models evaluating hypertension and home blood pressure monitoring adherence in a 2-group trajectory model (high adherence vs low adherence) as a sensitivity analysis**

|  | **Women with Hypertension**** | **Men with Hypertension**** |
| --- | --- | --- |
|  | High adherence# | High adherence# |
|  | aOR | aOR |
|  | (95% CI) | (95% CI) |
| **Unadjusted Model** | 1.39 (0.93, 2.08) | 1.02 (0.67, 1.56) |
| **Model 1*** | 1.13 (0.75, 1.72) | 0.69 (0.44, 1.10) |
| **Model 2**† | 1.14 (0.74, 1.75) | 0.67 (0.41, 1.08) |
| **Model 3**‡ | 1.22 (0.78, 1.89) | 0.76 (0.47, 1.22) |
| **Model 4**§ | 1.17 (0.76, 1.81) | 0.73 (0.46, 1.17) |
| **Model 5**\|\| | 1.24 (0.79, 1.95) | 0.81 (0.50, 1.32) |

* Model 1: adjusted for age

† Model 2: adjusted for age, income

‡ Model 3: adjusted for age, baseline systolic BP

§ Model 4: adjusted for age, anxiety, depression

|| Model 5: adjusted for age, anxiety, depression, baseline systolic BP

# Versus the “low” group

** Versus the same sex without hypertension
