# Supplementary material for: Patterns of adherence to home blood pressure monitoring among men and women in the Electronic Framingham Heart Study
Source: PLoS One. 2025 Dec 17;20(12):e0337284. doi: 10.1371/journal.pone.0337284 (PMC12711059; doi:10.1371/journal.pone.0337284)
Supplement: S2 Table — (DOCX) [file pone.0337284.s002.docx]

**Supporting information**

**S2 Table. Multivariable logistic regression models evaluating hypertension and home blood pressure monitoring adherence**

|  | **Participants with Hypertension**** | |
| --- | --- | --- |
|  | High adherence#  OR (95% CI) | Gradual decrease#  OR (95% CI) |
| **Unadjusted model** | **1.55 (1.08, 2.23)** | 1.06 (0.76, 1.49) |
| **Model 1*** | 1.09 (0.75, 1.60) | 0.94 (0.66, 1.33) |
| **Model 2**† | 1.05 (0.71, 1.55) | 0.89 (0.62, 1.28) |
| **Model 3**‡ | 1.18 (0.79, 1.77) | 0.92 (0.64, 1.31) |
| **Model 4**§ | 1.14 (0.77, 1.68) | 0.93 (0.65, 1.33) |
| **Model 5**\|\| | 1.23 (0.82, 1.86) | 0.91 (0.63, 1.32) |

* Model 1: adjusted for age

† Model 2: adjusted for age, income

‡ Model 3: adjusted for age, baseline systolic BP

§ Model 4: adjusted for age, anxiety, depression

|| Model 5: adjusted for age, anxiety, depression, baseline systolic BP

# Versus the “early discontinuation” group

** Versus participants without hypertension
