# Supplementary material for: Patterns of adherence to home blood pressure monitoring among men and women in the Electronic Framingham Heart Study
Source: PLoS One. 2025 Dec 17;20(12):e0337284. doi: 10.1371/journal.pone.0337284 (PMC12711059; doi:10.1371/journal.pone.0337284)
Supplement: S3 Table — (DOCX) [file pone.0337284.s003.docx]

**Supporting information**

**S3 Table.** **Home blood pressure monitoring adherence by sex and hypertension status**

|  | Early discontinuation  (N=428) | Gradual decrease (N=339) | High adherence  (N=222) | *P*-value |
| --- | --- | --- | --- | --- |
| **Sex distribution among participants with hypertension** | | | | |
| Women with hypertension | 42 (42.0) | 46 (55.4) | 38 (53.5) | 0.14 |
| Men with hypertension | 58 (58.0) | 37 (44.6) | 33 (46.5) |  |
| **Women stratified by hypertension status** | | | | |
| Women with hypertension | 42 (17.1) | 46 (22.0) | 38 (31.7) | **0.007** |
| Women without hypertension | 203 (82.9) | 163 (78.0) | 82 (68.3) |  |
| **Men stratified by hypertension status** | | | | |
| Men with hypertension | 58 (31.7) | 37 (28.5) | 33 (32.7) | 0.75 |
| Men without hypertension | 125 (68.3) | 93 (71.5) | 68 (67.3) |  |
